# Healthy lifestyle moderates the association between recent negative life events and depressive symptoms: a cross-sectional study

**DOI:** 10.3389/fpubh.2025.1575149

**Published:** 2025-05-19

**Authors:** Yang Yang, Yuhua Liao, Yanzhi Li, Huimin Zhang, Yifeng Liu, Guangduoji Shi, Jiejing Hao, Ruiying Chen, Ye Xu, Zhiyao Xin, Subinuer Yiming, Wanxin Wang, Lan Guo, Ciyong Lu, Beifang Fan

**Affiliations:** ^1^Department of Medical Statistics and Epidemiology, School of Public Health, Sun Yat-sen University, Guangzhou, China; ^2^Guangdong Provincial Key Laboratory of Food, Nutrition and Health, Sun Yat-sen University, Guangzhou, China; ^3^Department of Psychiatry, Shenzhen Nanshan Center for Chronic Disease Control, Shenzhen, China

**Keywords:** recent negative life events, overall lifestyle, depressive symptoms, moderating role, mental health

## Abstract

**Background:**

Both recent negative life events (RNLEs) and lifestyle factors were associated with depressive symptoms, but it is unclear whether adopting a healthy lifestyle can mitigate the association between RNLEs and depressive symptoms. We aim to explore the modifying role of adopting a healthy lifestyle in the association between RNLEs and depressive symptoms.

**Method:**

A cross-sectional study was conducted among 4,278 participants aged 18–70 years. RNLEs includes 12 common negative life events that have occurred in the past year, and were classified as low RNLEs (0 RNLEs), intermediate RNLEs (1–5 RNLEs), and high RNLEs (6–12 RNLEs). A healthy lifestyle score in adulthood was constructed as the sum of five modifiable lifestyle factors (i.e., smoking status, drinking status, regular physical activity, sleep duration, and living alone), and was classified as unfavorable [0–2 points], intermediate [3 points], and favorable [4–5 points] groups. Depressive symptoms were assessed using the 9-item Patient Health Questionnaire. A cut-off value of ≥5 was used to identify participants with depressive symptoms.

**Results:**

A total of 1,366 (31.9%) participants had depressive symptoms. Individuals with high RNLEs had a higher likelihood of depressive symptoms (odds ratio [OR] = 8.30, 95% confidence interval [95%CI]: 6.72–10.33, *p* < 0.001) compared to those with low RNLEs. The prevalence of depressive symptoms decreased with the adoption of more favorable lifestyle categories, with the lowest likelihood observed among individuals with a favorable lifestyle (0.35 [0.30–0.41]). The overall lifestyle was observed as significant moderating role on the association between RNLEs and depressive symptoms (OR, 95%CI: 0.61 [0.40–0.95]).

**Conclusion:**

In this cross-sectional study, healthy lifestyle act as a moderating role and adopting a greater number of healthy lifestyles was associated with a lower likelihood of depressive symptoms, and mitigated the association between RNLEs and depressive symptoms.

## Introduction

Depression is a prevalent mental disorder, affecting approximately 280 million individuals globally, with an annual increase in prevalence. The World Health Organization (WHO) has forecast that by 2030, depression will become the leading cause of global disease burden (1, 2). In China, the National Mental Health Survey indicates that the lifetime prevalence of depression among adults is 3.4%. Currently, there are approximately 95 million people suffering from depression in China, and approximately 280,000 suicides occur each year, with 40% of these individuals having depression (3). If left unaddressed, symptoms of depression, which are often considered a subhealth condition, can escalate into full-blown depression, posing significant threats to both mental and physical health (4).

Previous research has indicated that recent negative life events (RNLEs), such as loss of relatives, physical health problems, and financial troubles, functioning as a stressor, are a significant risk factor for depression (5–9). Importantly, unlike modifiable factors such as lifestyle, RNLEs, once occurred, are unmodifiable risk factors for depression, and their impacts may persist over a lifetime. Therefore, it is necessary to identify factors that could effectively mitigate the adverse influence of RNLEs on depression among adults.

Potential protective factors in the development process of depression have been extensively studied, and healthy lifestyle, such as regular physical activities and enough sleep duration, has received particular attention (10–12). Previous observational and intervention studies that have demonstrated that adherence to a healthy lifestyle is associated with a decreased risk of developing depression. Depression is often accompanied by over-activation of the HPA axis, leading to elevated cortisol levels (13). Chronic high cortisol levels are strongly associated with mood disorders. RNLEs can activate the HPA axis (14–16), and healthy lifestyles, such as physical exercise, can help regulate the function of the HPA axis and reduce the stress response (17). Physical exercise can promote neuroplasticity, improve mood through endorphin release, and regulate the immune system, all of which can counterbalance the negative effects of chronic HPA axis activation. Additionally, sufficient sleep and other positive lifestyle behaviors can further contribute to the stabilization of the HPA axis (18–20), reducing the long-term psychological and physiological consequences of stress exposure. In order to better intervene or reduce this association, it may be better to find a moderating variable that allows people to act on their own, and lifestyle is such a moderating variable, which is more practical. However, limited research has been conducted in this area (21). Besides, previous studies tended to use single variables to represent individual lifestyle levels, which only partially reflect overall lifestyle (22). However, lifestyle factors are interrelated, and few studies have built a healthy lifestyle score to reflect overall lifestyle and to evaluate its impact on the RNLEs in mental health.

To address these gaps in knowledge, we aimed to investigate whether a healthier overall lifestyle could mitigate the likelihood of depressive symptoms among those with RNLEs.

## Methods

### Study design and participants

This cross-sectional study was conducted in Guangdong Province, China, covering two distinct regions: Nanshan District (urban area) of Shenzhen City and Wengyuan County (rural area) of Shaoguan City. The study utilized baseline data from an ongoing longitudinal prospective cohort study of community-dwelling residents in mainland China. The recruitment process involved selecting adults aged 18 to 70 years from communities in both regions using a convenience sampling method. The personal information of the participants remained confidential all throughout the study. Because our data collection comprised only a single wave of baseline observations, a cross-sectional design was the only feasible approach and allowed us to estimate the prevalence of recent negative life events and depressive symptoms and examine their associations across subpopulations at one point in time.

The initial sample included 5,219 adults, of which 4,970 adults participated in the survey. At last, 4278 adults completed the whole set of questionnaires. The sample consists 2,588 (60.5%) female and 1,690 (39.5%) male adults. The age range was 18–70 years (Mean, 48.95; SD, 13.57).

### Assessment of depressive symptoms

Depressive symptoms were measured using the nine-item Patient Health Questionnaire (PHQ-9), ranging from 0 to 27. The PHQ-9 for the Chinese version has good internal consistency, reliability, and validity, with a Cronbach’s 0.91 (23). Various ratings describe the severity of depression symptoms, with 0–4 indicating no symptoms, 5–9 indicating mild symptoms, 10–14 indicating moderate symptoms, and 15–27 indicating severe symptoms (24). For this study, we focused on depressive symptoms rather than the disease, and considering that above 5 is the criterion for determining the presence of depressive symptoms, we set 5 as the cut-off score in this study (24). We separated the participants into two groups, with those scoring 0–4 as having no depression symptoms and those scoring 5 and above as having more than mild depressive symptoms.

### Assessment of recent negative life events

Participants reported whether or not they had experienced the following 12 recent negative life events over the past year: excessive fatigue (work, study, or daily life), death of a close family member (spouse or close relatives), family discord or marital problems (serious conflicts, separation, divorce, heartbreak, or difficulty in finding a partner), career changes (retirement, resignation, job transfer, transfer, or dropping out), economic difficulties (debt, losses, and theft), difficulties in career or study, troubles in relationships (superiors, colleagues, and neighbors), issues related to the future of children (difficulties in education, employment, or marriage), poor living environment (overcrowding, noise, and relocation), serious illness or injury to a close relative, and adverse changes in personal health, involving cases. To facilitate subsequent analysis and interpretation, we categorized RNLEs into three groups based on the number of exposures: “Low RNLEs,” “Intermediate RNLEs,” and “High RNLEs,” corresponding to 0, 1–5 and 6–12 RNLEs, respectively.

### Assessment of overall lifestyle

Since multiple lifestyles are interrelated and are associated with depressive symptoms, we constructed an overall lifestyle score including five modifiable lifestyle factors: smoking status, alcohol status, physical activity, sleep duration, and living alone according to previous studies (25, 26). We defined healthy and unhealthy levels for each lifestyle factor. Currently not smoking, currently not drinking, currently not living alone, optimal sleep (7–9 h/night) (27), and regular physical activity (moderate or vigorous physical activity ≥150 min/week) (28)were defined as healthy.

For each lifestyle factor, we assigned 1 point for a healthy level and 0 points for an unhealthy level. Thus, the overall lifestyle score was the sum of the points and ranged between 0 and 5, with higher scores indicating a healthier lifestyle. To balance the sample sizes and increase the statistical power, the overall lifestyle score was further categorized into three groups: unfavorable (0–2 points), intermediate (3 points), and favorable (4–5 points).

### Covariates

Covariates were obtained through questionnaires, including age, gender, region, household income, only child, educational level, and marital status. Household income was categorized into less than ¥30,000, ¥30,000 to 79,999, ¥80,000 to 149,999, ¥150,000 to 790,000, and more than ¥800,000. Education was classified into three levels: primary or below, junior or senior, and college or above. Marital status was grouped into single, married, and other status.

### Statistics analysis

Descriptive summaries are presented as means and standard deviations for continuous variables and as frequencies and percentages for categorical variables. Differences in each variable based on depressive symptoms (PHQ-9 ≥ 5) were evaluated by two-sample *t*-tests for continuous variables and Pearson chi-square tests for categorical variables.

Second, logistic regression analysis was used to examine the association of RNLEs and lifestyle with depressive symptoms among adults, and odds ratios (ORs) and 95% CIs for depressive symptoms were estimated. Two analytical models were created with the crude and adjusted models, including all covariates. Third, we assessed the prevalence of depressive symptoms associated with the joint categories of RNLEs (no, intermediate, and high) and overall lifestyle in adulthood (unfavorable, intermediate, and favorable), using the low RNLEs and favorable lifestyle as the reference. Fourth, we compared the associations of adherence to a favorable or intermediate lifestyle in adulthood with the prevalence of depressive symptoms within each RNLEs category. Finally, subgroup analyses were performed according to the age category (18–44 years, 45–59 years, and ≥60 years), gender (women and men), and region (urban and rural).

All statistical analyses were performed using R software (4.3.0). We considered two-sided *p*-values <0.05 as statistically significant.

## Results

### Population characteristics

Table 1 shows the characteristics of the study population. Of the 4,278 participants (39.5% men, mean age, 49.0 years) included in the analyses, 1,366 (31.9%) had depressive symptoms. A total of 1,141 (26.7%) participants reported not experiencing RNLEs (low RNLEs), 1724 (40.3%) participants reported experiencing 2–5 types of RNLEs (intermediate NLEs), and 1,413 (33.0%) participants reported experiencing 6 or more types of RNLEs (high RNLEs). Participants with depressive symptoms were more likely to be younger, female, urban, not-only child, single, and with lower household income and lower education level (all *p* values <0.001). In addition, RNLEs, drinking, smoking, inappropriate sleep duration, living alone, and inadequate physical activity were more prevalent among adults with depressive symptoms (all *p* values <0.001).

**Table 1:** Demographic characteristics of participants.

Variables	Total (*n* = 4,278)	Depressive symptoms		*p*-value
		Yes (*n* = 1,366)	No (*n* = 2,912)	
Age, mean (SD), year	49.0 (13.6)	46.2 (12.6)	49.4 (11.5)	<0.001
Gender				<0.001
Woman	2,588 (60.5)	872 (63.8)	1716 (58.9)	
Man	1,690 (39.5)	494 (36.2)	1,196 (41.1)	
Region				<0.001
Urban	2,442 (57.1)	1,108 (81.1)	1,334 (45.8)	
Rural	1836 (42.9)	258 (18.9)	1,578 (54.2)	
Household income (¥)				<0.001
<30,000	1956 (45.7)	515 (37.7)	1,441 (49.4)	
30,000–79,999	967 (22.6)	295 (21.6)	672 (23.1)	
80,000–149,999	817 (19.1)	326 (23.9)	492 (16.9)	
150,000–790,000	504 (11.8)	218 (16.0)	286 (9.8)	
≥800,000	34 (0.8)	12 (0.9)	22 (0.8)	
Only Child				<0.001
Yes	401 (9.4)	201 (14.7)	200 (6.9)	
No	3,877 (90.6)	1,165 (85.3)	2,712 (93.1)	
Education level				<0.001
Primary or below	857 (20.0)	190 (13.9)	667 (22.9)	
Junior or senior	1,665 (38.9)	382 (28.0)	1,283 (44.1)	
College or above	1756 (41.0)	794 (58.1)	962 (33.0)	
Marital status				<0.001
Single	856 (20.0)	474 (34.7)	382 (13.1)	
Married	3,199 (74.8)	814 (59.6)	2,385 (81.9)	
Other status	223 (5.2)	78 (5.7)	145 (5.0)	
RNLEs^a^				<0.001
Low RNLEs	1,141 (26.7)	129 (9.4)	1,012 (34.8)	
Intermediate RNLEs	1724 (40.3)	509 (37.3)	1,215 (41.7)	
High RNLEs	1,413 (33.0)	728 (53.3)	685 (23.5)	
Drinking status				<0.001
Yes	2,218 (51.8)	808 (59.2)	1,410 (48.4)	
No	2060 (48.2)	558 (40.8)	1,502 (51.6)	
Smoking status				<0.001
Yes	636 (14.9)	205 (15.0)	431 (14.8)	
No	3,642 (85.1)	1,161 (85.0)	2,481 (85.2)	
Sleep duration				<0.001
7–9 h	1,215 (28.4)	325 (23.8)	890 (30.6)	
<7 h or >9 h	3,063 (71.6)	1,041 (76.2)	2022 (69.4)	
Living alone				<0.001
Yes	384 (9.0)	167 (12.2)	217 (7.5)	
No	3,894 (91.1)	1,199 (87.8)	2,695 (92.5)	
Physical activity				<0.001
MPA or VPA ≥ 150 min/week^b^	2,549 (59.6)	740 (54.2)	1809 (62.1)	
MPA or VPA < 150 min/week	1729 (40.4)	626 (45.8)	1,103 (37.9)	

Unless otherwise indicated, data are expressed as NO. (%) of participants.

a Low RNLEs: no recent negative life events, Intermediate RNLEs: 1–5 types of recent negative life events, High RNLEs: 6–12 types of recent negative life events.

b MPA: moderate physical activity, VPA: vigorous physical activity.

### Associations of RNLEs with depressive symptoms

**Figure 1:**
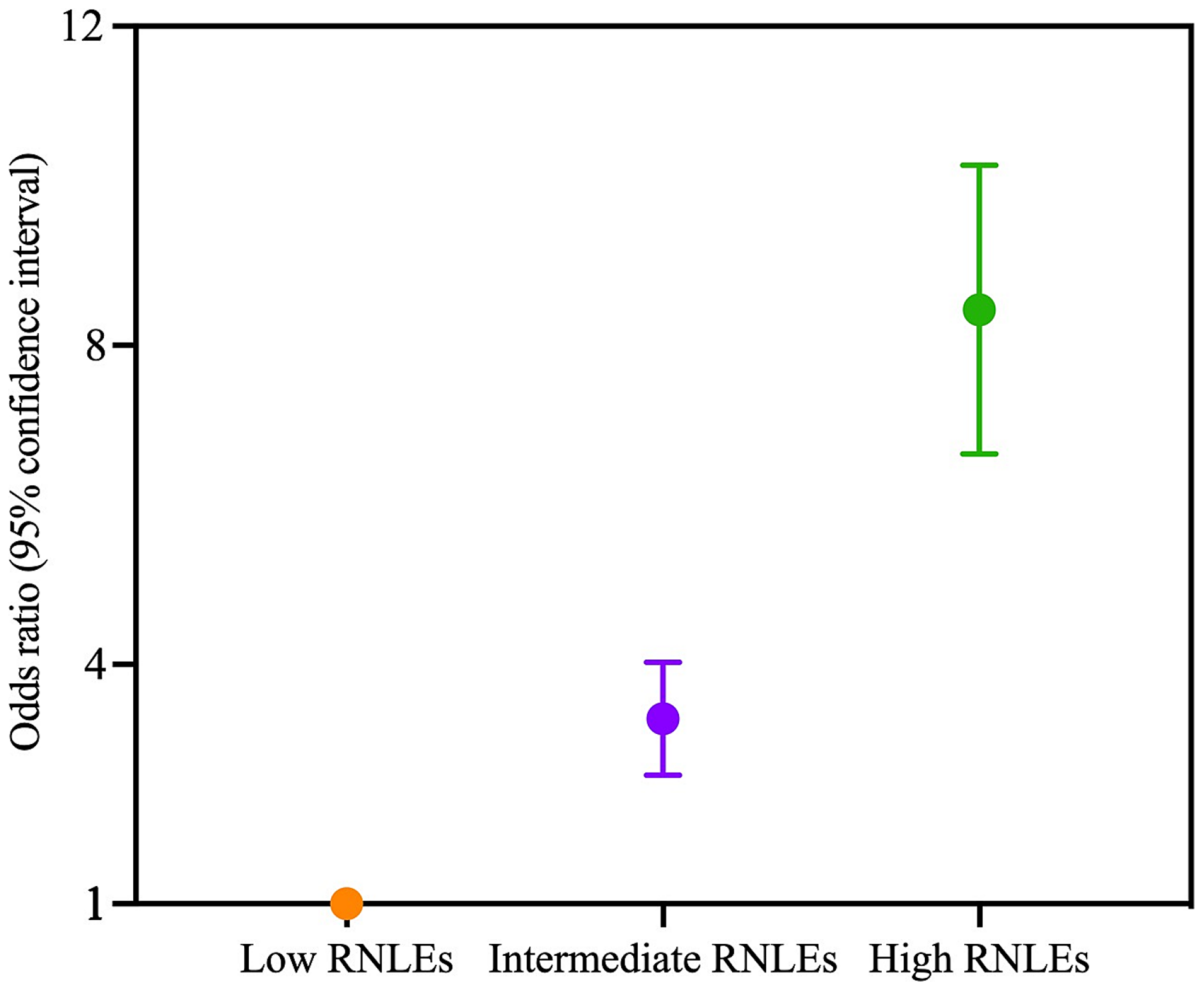
Association of recent negative life events (RNLEs) with the odds of depressive symptoms. Participants were divided into three exposure groups according to the number of recent negative life events (RNLEs) (low [0 RNLEs], intermediate [2–5 RNLEs], high [6 or more RNLEs]). Odds ratio (OR) with 95% confidence interval (CI) for each group was compared with the low RNLEs group. Points represent the ORs, and error bars show the 95% CIs. The model was adjusted for age, gender, region, only child, education level, household income, marital status, and overall lifestyle in adulthood.

Figure 1 shows that the prevalence of depressive symptoms increased significantly across three RNLEs categories. Compared with individuals with low RNLEs (Table 2), those with high RNLEs had a higher association with depressive symptoms (OR, 95%CI: 8.30, 6.72–10.33) (Table 2). An increase in the number of RNLEs was more strongly associated with depressive symptoms in a dose–response manner (adjusted for all covariates, all *p* values for overall association <0.05, Supplementary Figure S1).

**Table 2:** Association of RNLEs and overall lifestyle with depressive symptom.

Variable	*N* (%)	Crude *OR* (95%*CI*)	Adjusted^a^ *OR* (95% *CI*)
RNLEs			
Low RNLEs	129/1141 (11.3%)	Ref.	Ref.
Intermediate RNLEs	509/1724 (29.5%)	3.15 (2.54–3.93)^***^	3.26 (2.65–4.06)^***^
High RNLEs	728/1413 (51.5%)	7.76 (6.26–9.68)^***^	8.30 (6.72–10.33)^***^
Overall lifestyle			
Unfavorable lifestyle	568/1310 (43.4%)	Ref.	Ref.
Intermediate lifestyle	427/1148 (37.2%)	0.82 (0.69–0.98)^*^	0.78 (0.66–0.92)^***^
Favorable lifestyle	371/1820 (20.4%)	0.39 (0.33–0.47)^***^	0.35 (0.30–0.41)^***^

RNLEs, recent negative life events; OR, odds ratio, CI, confidence interval.

a Adjusted for age, gender, region, household income, only child, educational level, and marital status. In the two adjusted model, RNLEs and overall lifestyle were mutually adjusted for each other.

^*^ *p* < 0.05; ^**^ *p* < 0.001; ^***^ *p* < 0.0001.

### Associations of lifestyle with depressive symptoms

**Figure 2:**
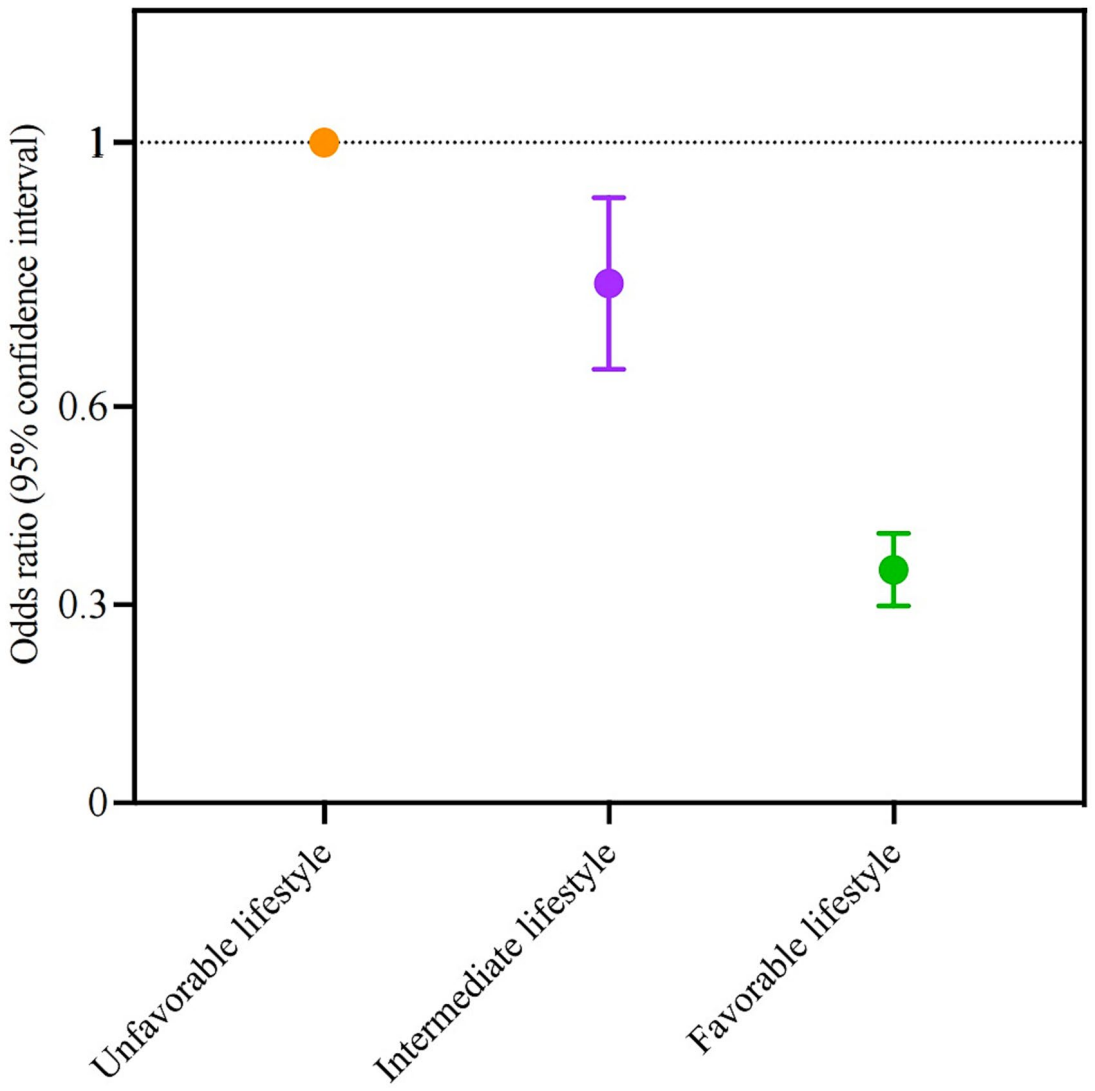
Association of overall lifestyle in adulthood with the odds ratio of depressive symptoms. Participants were divided into three groups according to the number of healthy lifestyle factors in adulthood (unfavorable [0–2 point], intermediate [3 points], favorable [4–5 points]). Participants with an unfavorable lifestyle were regarded as the reference group. Points represent the odds ratios, and error bars show the 95% confidence intervals. The model was adjusted for age, gender, region, only child, education level, household income, marital status, and negative life events in adulthood.

The prevalence of depressive symptoms significantly decreased as the lifestyle category became more favorable (Figure 2). Participants with a favorable lifestyle were less tightly associated with depressive symptoms (0.35, 0.30–0.41). There was also a dose–response association between healthy lifestyle scores and depressive symptoms (adjusted for all covariates, *p* values <0.05 when healthy lifestyle score >1, Supplementary Figure S2).

### Joint analysis of overall lifestyle and RNLEs with depressive symptoms

Supplementary Figure S4 shows the joint associations of overall lifestyle and recent negative life events with depressive symptoms. There was an upward trend in the prevalence of depressive symptoms with higher RNLEs and an increasingly unhealthy lifestyle. The overall lifestyle was observed as significant moderating role on the association between RNLEs and depressive symptoms (OR, 95%CI: 0.61 [0.40–0.95]). There was a significant interaction effect between RNLEs and overall lifestyle on depressive symptoms (*p* value <0.0001).

**Figure 3:**
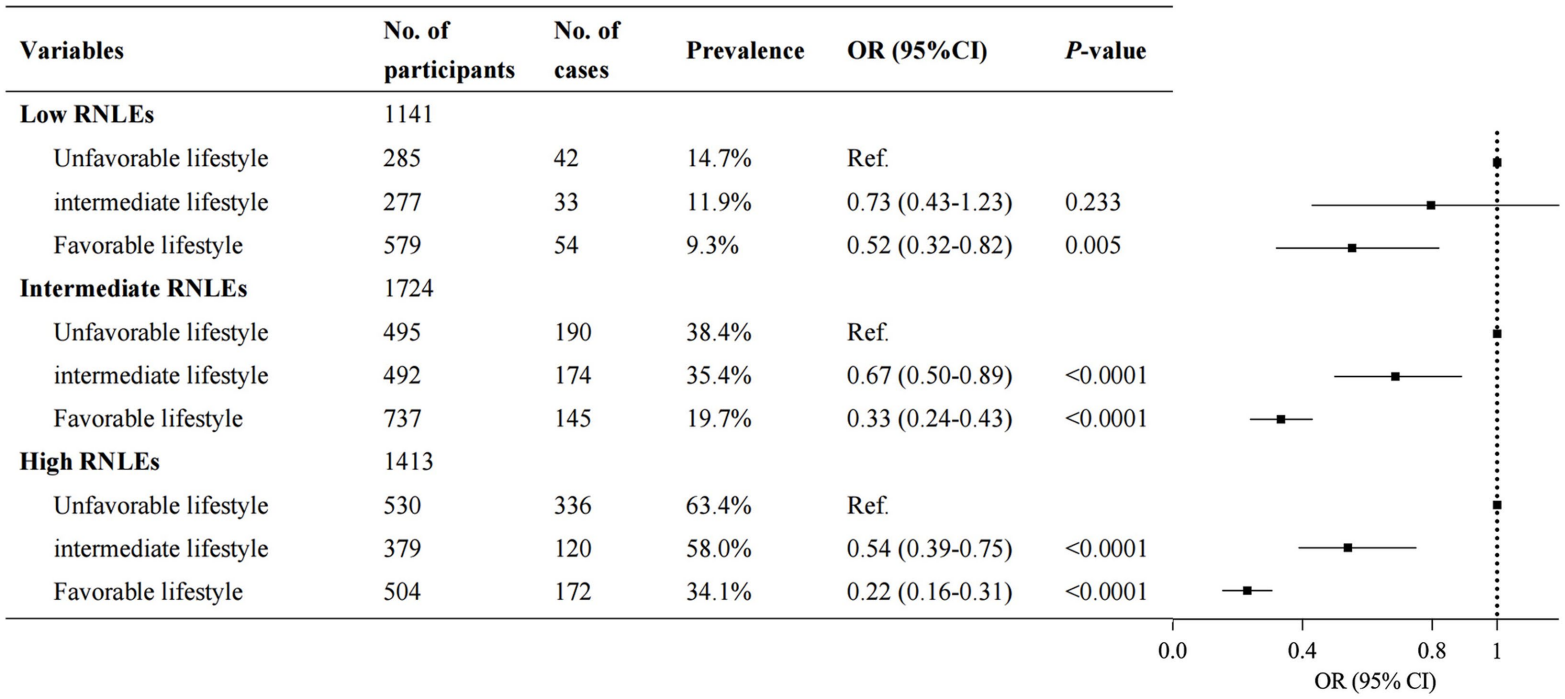
Association between overall lifestyle in adulthood and the odds ratio of depressive symptoms within each recent negative life events (RNLEs) category. Participants with an unfavorable lifestyle were considered as the reference group within each RNLEs category. Points represent the odds ratios, and error bars show the 95% confidence intervals. The model was adjusted for age, gender, region, only child, education level, household income, and marital status.

Within each stratum of RNLEs (low, intermediate, high), we observed a significant reduction in the proportion of depressive symptoms in participants who adopted a favorable or intermediate lifestyle in adulthood compared to those with an unfavorable lifestyle (Figure 3). In participants with high RNLEs, the association between RNLEs and depressive symptoms dropped by 78% (OR, 95%CI: 0.22 [0.16–0.31]) with a favorable lifestyle compared to those with an unfavorable lifestyle.

### Subgroup and sensitivity analysis

Subgroup analyses by age, gender, and region were similar to our primary analyses (Supplementary Table S1). The associations of RNLEs and overall lifestyle with depressive symptoms did not materially change when using 10 points as the cut-off of PHQ-9 (Supplementary Table S2).

## Discussion

### Principal findings

In this cross-sectional study, we found that a healthier lifestyle and fewer recent negative life events were associated with a lower prevalence of depressive symptoms. However, our key finding was that having a favorable lifestyle in adulthood notably decreased the association between depressive symptoms and RNLEs irrespective of the RNLEs exposure category.

### Comparison with previous studies

Consistent with our findings, prior studies suggested RNLEs play an important role in the prevalence of depressive symptoms. Individuals with more RNLEs are exposed to more stress, social challenges, and adversity, but have limited approaches to cope with these events, and thus are more likely to suffer from depression (29). Similarly, a meta-analysis of 30 cohort studies with 25,154 college students reported a 16% increased risk of depressive symptoms with RNLEs (30). Another meta-analysis of 11 studies with 31,528 older Chinese adults found RNLEs as significant risk factors (OR, 95%CI: 2.43 [1.87.2.98]). By comparison, our results also show that different types of RNLEs, their aggregate number, and categories were independently associated with an increased risk of depressive symptoms. Moreover, our results highlight the importance of early screening for individuals with RNLEs.

Meta-analysis and systematic analysis have indicated that healthy lifestyles are associated with the alleviation of depressive symptoms (25, 31). A systematic review and meta-analysis of 15 prospective studies including more than 2 million person-years showed an inverse curvilinear association between physical activity and incident depression, with greater differences in risk at lower exposure levels (32). A cross-sectional study comprising 176,794 individuals aged 19 years and over suggest poor sleep quality may contribute to depressive symptoms (33). These results were consistent with our findings. Further, we found that currently no drinking and no smoking were associated with a reduced prevalence of depressive symptoms. Several previous studies have also investigated the association of smoking and alcohol assumption with depression, but the results were inconsistent. For instance, a community-based survey showed that having an alcohol use disorder at least doubles the odds of depressive, anxiety, and other non-substance-use disorders (34). However, a cohort study of 12,686 individuals suggest that moderate alcohol consumption may be associated with a reduced risk of depression (35). A systematic review of 110 original research articles, eight meta-analyses and reviews, and four reports and websites revealed no cause-effect relationship (36). In addition, prior studies have found that living alone is a risk factor for depressive symptoms in both older and younger people. A prospective study of 3,011 older adults in South Korea showed a significant increase in depressive symptoms during the first, second, and third years of living alone compared with living with others (37). According to new U.S. Census Bureau data, younger adults living alone were more likely than older adults living alone to report depressive symptoms (38).

However, limited evidence is available on whether a healthy lifestyle moderates the association between recent negative life events and depressive symptoms among adults. A cohort study of 3,597 adults aged 25 years or older suggested that poor sleep has the ability to compound the effects of recent negative life events (21). Besides, a longitudinal study of 316 adolescents in America presented sleep efficiency as a modulator of stress-related risk (22). Both results support our findings to some extent. Notably, most previous studies focused the single lifestyle factors whereas we included 3 dimensions of overall lifestyle, which would have a more comprehensive assessment. Additionally, our study is the first to show a significant reduction in the association between depressive symptoms and different categories of RNLEs through adopting a healthy lifestyle in adulthood.

Subgroup analyses indicates that, after stratifying by age, gender, and region, a favorable lifestyle continues to attenuate the association between RNLEs and depressive symptoms in both the intermediate and high RNLEs groups. Moreover, this mitigating effect is particularly pronounced among participants under 60 years old, women, and urban residents. These findings indicate that promoting a favorable lifestyle may yield greater benefits for these populations.

These findings reinforce the message of policymakers and healthcare practitioners to promote healthy lifestyles (smoking and drinking cessation, engaging in appropriate physical activity, adequate sleep duration, and avoiding living alone) across the population but also highlight that those with high RNLEs may benefit most.

### Strengths and limitations

To our knowledge, this is the first study to investigate the joint association between RNLEs and overall lifestyles with depressive symptoms among adults. In addition, we established overall healthy lifestyle scores and leveled RNLEs to comprehensively evaluate the stratified and joint associations of a healthy lifestyle and negative life events with depressive symptoms. We also conducted a series of sensitivity analyses to show the robustness of the findings and evaluated the moderation within different age, gender, and region groups.

This study has several limitations. First, because data collection was limited to a single baseline wave of cross-sectional observations, we were unable to infer causal relationships. Second, our measures were based on the participants’ self-report, so reporting bias and recall bias was inevitable. Finally, the sample was from Guangdong Province, China, so caution should be exercised when applying results in other regions.

## Conclusion

In this cross-sectional study among adults, adopting to a greater number of healthy lifestyles was associated with a lower likelihood of depressive symptoms, and mitigated the association between RNLEs and depressive symptoms. Our findings underscore the potential role of adopting a healthy lifestyle in adulthood for those with RNLEs to reduce their risk of depression.

## Data Availability

The raw data supporting the conclusions of this article will be made available by the authors, without undue reservation.

## References

[1] Hu T Zhao X Wu M Li Z Luo L Yang C. Prevalence of depression in older adults: a systematic review and meta-analysis. Psychiatry Res. (2022) 311:114511. doi: 10.1016/j.psychres.2022.114511, PMID: 35316691

[2] Thapar A Eyre O Patel V Brent D. Depression in young people. Lancet. (2022) 400:617–31. doi: 10.1016/S0140-6736(22)01012-1, PMID: 35940184

[3] Huang Y Wang Y Wang H Liu Z Yu X Yan J. Prevalence of mental disorders in China: a cross-sectional epidemiological study. Lancet Psychiatry. (2019) 6:211–24. doi: 10.1016/S2215-0366(18)30511-X 30792114

[4] Holtfreter K Reisig MD Turanovic JJ. Depression and infrequent participation in social activities among older adults: the moderating role of high-quality familial ties. Aging Ment Health. (2017) 21:379–88. doi: 10.1080/13607863.2015.1099036, PMID: 26471453

[5] Maciejewski D Van Sprang E Spinhoven P Penninx B. Longitudinal associations between negative life events and depressive symptoms—a 9-year longitudinal study on between-person and within-person effects and the role of family history. J Pers Soc Psychol. (2021) 121:707–21. doi: 10.1037/pspp0000381, PMID: 33507780

[6] Ji L Chen C Hou B Ren D Yuan F Liu L. A study of negative life events driven depressive symptoms and academic engagement in Chinese college students. Sci Rep. (2021) 11:17160. doi: 10.1038/s41598-021-96768-9, PMID: 34433874 PMC8387499

[7] Sun XJ Niu GF You ZQ Zhou ZK Tang Y. Gender, negative life events and coping on different stages of depression severity: a cross-sectional study among Chinese university students. J Affect Disord. (2017) 209:177–81. doi: 10.1016/j.jad.2016.11.025, PMID: 27923194

[8] Asselmann E Wittchen HU Lieb R Höfler M Beesdo-Baum K. Danger and loss events and the incidence of anxiety and depressive disorders: a prospective-longitudinal community study of adolescents and young adults. Psychol Med. (2015) 45:153–63. doi: 10.1017/S0033291714001160, PMID: 25065411

[9] Xie Y Ma D Duan Y Cao J Wei J. The association among negative life events, alexithymia, and depressive symptoms in a psychosomatic outpatient sample. BMC Psychiatry. (2024) 24:451. doi: 10.1186/s12888-024-05902-0, PMID: 38890601 PMC11186062

[10] Aneshensel CS Huba GJ. Depression, alcohol use, and smoking over one year: a four-wave longitudinal causal model. J Abnorm Psychol. (1983) 92:134–50. doi: 10.1037//0021-843x.92.2.134, PMID: 6863729

[11] Han B Volkow ND Blanco C Tipperman D Einstein EB Compton WM. Trends in prevalence of cigarette smoking among US Adults with major depression or substance use disorders, 2006-2019. JAMA. (2022) 327:1566–76. doi: 10.1001/jama.2022.4790, PMID: 35471512 PMC9044114

[12] Zhai L Zhang H Zhang D. Sleep duration and depression among adults: a meta-analysis of prospective studies. Depress Anxiety. (2015) 32:664–70. doi: 10.1002/da.22386, PMID: 26047492

[13] Kupfer DJ Frank E Phillips ML. Major depressive disorder: new clinical, neurobiological, and treatment perspectives. Lancet. (2012) 379:1045–55. doi: 10.1016/S0140-6736(11)60602-8, PMID: 22189047 PMC3397431

[14] van Bodegom M Homberg JR Henckens MJAG. Modulation of the hypothalamic-pituitary-adrenal Axis by early life stress exposure. Front Cell Neurosci. (2017) 11:87. doi: 10.3389/fncel.2017.00087, PMID: 28469557 PMC5395581

[15] Dickerson SS Kemeny ME. Acute stressors and cortisol responses: a theoretical integration and synthesis of laboratory research. Psychol Bull. (2004) 130:355–91. doi: 10.1037/0033-2909.130.3.355, PMID: 15122924

[16] Ehlert U Gaab J Heinrichs M. Psychoneuroendocrinological contributions to the etiology of depression, posttraumatic stress disorder, and stress-related bodily disorders: the role of the hypothalamus-pituitary-adrenal axis. Biol Psychol. (2001) 57:141–52. doi: 10.1016/s0301-0511(01)00092-8, PMID: 11454437

[17] Kandola A Ashdown-Franks G Hendrikse J Sabiston CM Stubbs B. Physical activity and depression: towards understanding the antidepressant mechanisms of physical activity. Neurosci Biobehav Rev. (2019) 107:525–39. doi: 10.1016/j.neubiorev.2019.09.040, PMID: 31586447

[18] Vgontzas AN Bixler EO Lin HM Prolo P Mastorakos G Vela-Bueno A. Chronic insomnia is associated with nyctohemeral activation of the hypothalamic-pituitary-adrenal axis: clinical implications. J Clin Endocrinol Metab. (2001) 86:3787–94. doi: 10.1210/jcem.86.8.7778, PMID: 11502812

[19] Wand GS Dobs AS. Alterations in the hypothalamic-pituitary-adrenal axis in actively drinking alcoholics. J Clin Endocrinol Metab. (1991) 72:1290–5. doi: 10.1210/jcem-72-6-1290, PMID: 2026749

[20] Mendelson JH Goletiani N Sholar MB Siegel AJ Mello NK. Effects of smoking successive low- and high-nicotine cigarettes on hypothalamic-pituitary-adrenal axis hormones and mood in men. Neuropsychopharmacology. (2008) 33:749–60. doi: 10.1038/sj.npp.1301455, PMID: 17507912

[21] Leggett A Burgard S Zivin K. The impact of sleep disturbance on the association between stressful life events and depressive symptoms. J Gerontol B Psychol Sci Soc Sci. (2016) 71:118–28. doi: 10.1093/geronb/gbv072, PMID: 26329114 PMC4861642

[22] Chiang JJ Kim JJ Almeida DM Bower JE Dahl RE Irwin MR. Sleep efficiency modulates associations between family stress and adolescent depressive symptoms and negative affect. J Adolesc Health. (2017) 61:501–7. doi: 10.1016/j.jadohealth.2017.04.011, PMID: 28729144 PMC5712225

[23] Kroenke K Spitzer RL Williams JB. The PHQ-9: validity of a brief depression severity measure. J Gen Intern Med. (2001) 16:606–13. doi: 10.1046/j.1525-1497.2001.016009606.x, PMID: 11556941 PMC1495268

[24] Wang W Bian Q Zhao Y Li X Wang W du J. Reliability and validity of the Chinese version of the patient health questionnaire (PHQ-9) in the general population. Gen Hosp Psychiatry. (2014) 36:539–44. doi: 10.1016/j.genhosppsych.2014.05.021, PMID: 25023953

[25] Wang X Arafa A Liu K Eshak ES Hu Y Dong JY. Combined healthy lifestyle and depressive symptoms: a meta-analysis of observational studies. J Affect Disord. (2021) 289:144–50. doi: 10.1016/j.jad.2021.04.030 33979724

[26] Zheng G Zhou B Fang Z Jing C Zhu S Liu M. Living alone and the risk of depressive symptoms: a cross-sectional and cohort analysis based on the China health and retirement longitudinal study. BMC Psychiatry. (2023) 23:853. doi: 10.1186/s12888-023-05370-y, PMID: 37978367 PMC10655346

[27] Baranwal N Yu PK Siegel NS. Sleep physiology, pathophysiology, and sleep hygiene. Prog Cardiovasc Dis. (2023) 77:59–69. doi: 10.1016/j.pcad.2023.02.005 36841492

[28] Yang D Yang M Bai J Ma Y Yu C. Association between physical activity intensity and the risk for depression among Adults from the National Health and nutrition examination survey 2007-2018. Front Aging Neurosci. (2022) 14:844414. doi: 10.3389/fnagi.2022.844414, PMID: 35711909 PMC9197339

[29] Caspi A Sugden K Moffitt TE Taylor A Craig IW Harrington HL. Influence of life stress on depression: moderation by a polymorphism in the 5-HTT gene. Science. (2003) 301:386–9. doi: 10.1126/science.1083968 12869766

[30] Liu Y Zhang N Bao G Huang Y Ji B Wu Y. Predictors of depressive symptoms in college students: A systematic review and meta-analysis of cohort studies. J Affect Disord. (2019) 244:196–208. doi: 10.1016/j.jad.2018.10.084, PMID: 30352363

[31] Zhang X Zhang L Liu Y Lin Y Yang X Gong L. The relationship between unhealthy lifestyle patterns and depressive symptoms among residents in Beijing, China: a community-based cross-sectional study. Front Public Health. (2023) 11:1055209. doi: 10.3389/fpubh.2023.1055209, PMID: 37124807 PMC10132209

[32] Pearce M Garcia L Abbas A Strain T Schuch FB Golubic R. Association between physical activity and risk of depression: a systematic review and Meta-analysis. JAMA Psychiatry. (2022) 79:550–9. doi: 10.1001/jamapsychiatry.2022.0609, PMID: 35416941 PMC9008579

[33] Joo HJ Kwon KA Shin J Park S Jang SI. Association between sleep quality and depressive symptoms. J Affect Disord. (2022) 310:258–65. doi: 10.1016/j.jad.2022.05.004 35545156

[34] Hasin DS Goodwin RD Stinson FS Grant BF. Epidemiology of major depressive disorder: results from the National Epidemiologic Survey on alcoholism and related conditions. Arch Gen Psychiatry. (2005) 62:1097–106. doi: 10.1001/archpsyc.62.10.1097, PMID: 16203955

[35] Visontay R Mewton L Slade T Aris IM Sunderland M. Moderate alcohol consumption and depression: a marginal structural model approach promoting causal inference. Am J Psychiatry. (2023) 180:209–17. doi: 10.1176/appi.ajp.22010043, PMID: 36651625

[36] Farooqui M Shoaib S Afaq H Quadri S Zaina F Baig A. Bidirectionality of smoking and depression in adolescents: a systematic review. Trends Psychiatry Psychother. (2023) 45:e20210429. doi: 10.47626/2237-6089-2021-0429, PMID: 35738567 PMC10416256

[37] Koo JH Son N Yoo KB. Relationship between the living-alone period and depressive symptoms among the elderly. Arch Gerontol Geriatr. (2021) 94:104341. doi: 10.1016/j.archger.2021.104341, PMID: 33497913

[38] United States Census Bureau. Household pulse survey data. Census.gov. (2024). Available online at: https://www.census.gov/programs-surveys/household-pulse-survey/data.html (accessed September 29, 2024).

