# Supplementary material for: Healthy lifestyle moderates the association between recent negative life events and depressive symptoms: a cross-sectional study
Source: Front Public Health. 2025 May 19;13:1575149. doi: 10.3389/fpubh.2025.1575149 (PMC12127182; doi:10.3389/fpubh.2025.1575149)
Supplement: Supplementary file 1 [file Supplementary_file_1.docx]

**Supplementary Materials**

**Title:** Healthy lifestyle moderates the association between recent negative life events and depressive symptoms: a cross-sectional study

[Table S1 Subgroup analyses for the association of lifestyle categories with depressive symptoms within each category of recent negative life events 1](#_Toc1773101354)

[Table S2 Association of RNLEs and overall lifestyle with depressive symptoms (PHQ-9≥10) 3](#_Toc2031370906)

[Figure S1 Dose-relationship of the number of RNLEs and odds ratio of depressive symptoms. 4](#_Toc555797136)

[Figure S2 Dose-relationship of the number of healthy lifestyle and odds ratio of depressive symptoms. 5](#_Toc1876083949)

[Figure S3 Association between recent negative life events (RNLEs) and odds ratio of depressive symptoms within each overall lifestyle category. 6](#_Toc1988025589)

[Figure S4 Joint association of recent negative life events (RNLEs) and overall lifestyle in adulthood with odds ratio of depressive symptoms. 7](#_Toc48010650)

# Table S1 Subgroup analyses for the association of lifestyle categories with depressive symptoms within each category of recent negative life events

| Lifestyle categories | Age | | | Gender | |  | Region | |
| --- | --- | --- | --- | --- | --- | --- | --- | --- |
|  | <60 years | ≥60 years |  | Women | Men |  | Urban | Rural |
| Low RNLEs |  |  |  |  |  |  |  |  |
| Unfavorable lifestyle | Ref. | Ref. |  | Ref. | Ref. |  | Ref. | Ref. |
| Intermediate lifestyle | 0.62 (0.36-1.08) | 0.89 (0.67-1.41) |  | 0.92 (0.47-1.81) | 0.91 (0.44-1.87) |  | 0.72 (0.41-1.27) | 0.60 (0.18-2.00) |
| Favorable lifestyle | 0.50 (0.31-0.82) | 0.63 (0.41-0.89) |  | 0.46 (0.25-0.85) | 0.45 (0.18-1.13) |  | 0.60 (0.30-1.00) | 0.23 (0.07-0.75) |
| Intermediate RNLEs |  |  |  |  |  |  |  |  |
| Unfavorable lifestyle | Ref. | Ref. |  | Ref. | Ref. |  | Ref. | Ref. |
| Intermediate lifestyle | 0.71 (0.52-0.97) | 0.71 (0.47-1.08) |  | 0.64 (0.44-0.92) | 0.67 (0.43-1.06) |  | 0.59 (0.42-0.84) | 0.82 (0.50-1.34) |
| Favorable lifestyle | 0.33 (0.24-0.45) | 0.36 (0.24-0.54) |  | 0.29 (0.21-0.42) | 0.37 (0.23-0.61) |  | 0.30 (0.21-0.43) | 0.34 (0.21-0.56) |
| High RNLEs |  |  |  |  |  |  |  |  |
| Unfavorable lifestyle | Ref. | Ref. |  | Ref. | Ref. |  | Ref. | Ref. |
| Intermediate lifestyle | 0.51 (0.36-0.72) | 0.62 (0.21-1.85) |  | 0.47 (0.29-0.75) | 0.55 (0.33-0.89) |  | 0.41 (0.26-0.65) | 0.68 (0.41-1.11) |
| Favorable lifestyle | 0.20 (0.14-0.28) | 0.34 (0.12-0.98) |  | 0.19 (0.12-0.29) | 0.27 (0.15-0.47) |  | 0.20 (0.13-0.32) | 0.21 (0.12-0.34) |

Data were presented as odds ratios (95% confidence intervals). Participants with an unfavorable lifestyle were regarded as the reference group. All models were adjusted for age, gender, region, only child, education level, household income, and marital status. RNLEs: recent negative life events.

# Table S2 Association of RNLEs and overall lifestyle with depressive symptoms (PHQ-9≥10)

| Variable | Total No. of participants | No.1of cases | Prevalence |  | Model 1^a^ | |  | Model 2^b^ | | |
| --- | --- | --- | --- | --- | --- | --- | --- | --- | --- | --- |
|  |  |  |  |  | OR (95%CI) | *P*-value |  |  | OR (95% CI) | *P*-value |
| **RNLEs** |  |  |  |  |  |  |  |  |  |  |
| Low RNLEs | 1141 | 32 | 2.8% |  | Ref. |  |  |  | Ref. |  |
| Intermediate RNLEs | 1724 | 104 | 6.0% |  | 2.07 (1.40-3.15) | <0.001 |  |  | 2.01 (1.34-3.08) | <0.001 |
| High RNLEs | 1413 | 261 | 18.5% |  | 8.67 (5.87-12.81) | <0.001 |  |  | 7.09 (4.88-10.60) | <0.001 |
| **Overall lifestyle** |  |  |  |  |  |  |  |  |  |  |
| Unfavorable lifestyle | 1310 | 173 | 13.2 |  | Ref. |  |  |  | Ref. |  |
| Intermediate lifestyle | 1148 | 148 | 12.9% |  | 0.80 (0.68-1.13) | 0.309 |  |  | 0.95 (0.73-1.24) | 0.721 |
| Favorable lifestyle | 1820 | 76 | 4.2% |  | 0.34 (0.26-0.45) | <0.001 |  |  | 0.35 (0.26-0.47) | <0.001 |

A score of 10 was used as a cut-off value for depressive symptoms in this analysis.

^a^Model 1 was adjusted for age, gender, region, household income, only child, educational level, and marital status.

^b^Model 2 was additionally adjusted for overall lifestyle for RNLEs, and for RNLEs additionally for overall lifestyle.

RNLEs: recent negative life events; OR: odds ratio; CI: confidence interval.


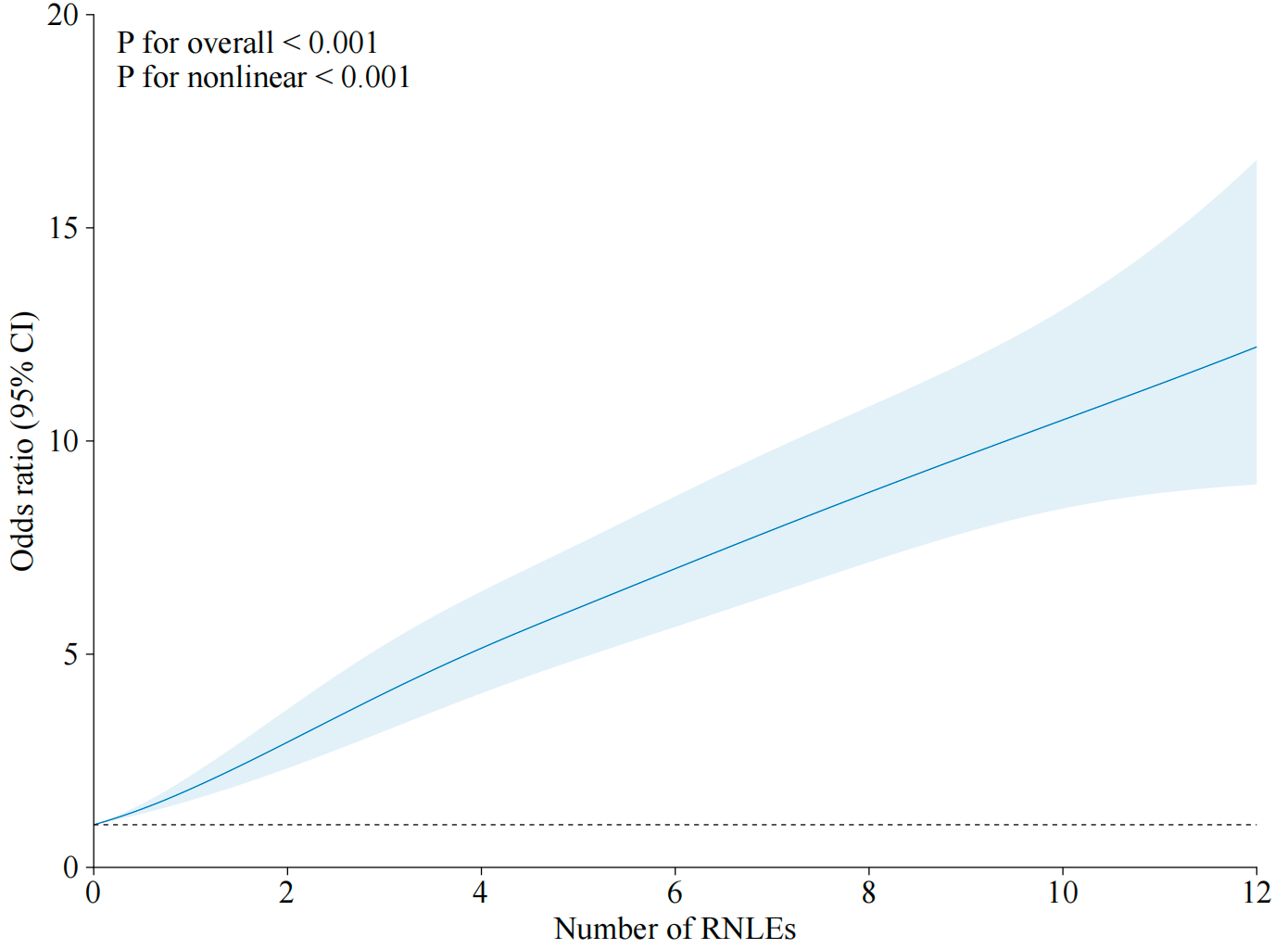


# Figure S1 Dose-relationship of the number of RNLEs and odds ratio of depressive symptoms.

Graphs show ORs for depressive symptoms according to RNLEs adjusted for age, gender, region, household income, only child, educational level, marital status, and overall lifestyle. Data were fitted by a logistic regression model, and the model was conducted with 4 knots at the 5th, 35th, 65th, 95th percentiles of recentevent. Solid lines indicate ORs, and shadow shape indicate 95% CIs.

OR: odds ratio; CI: confidence interval.


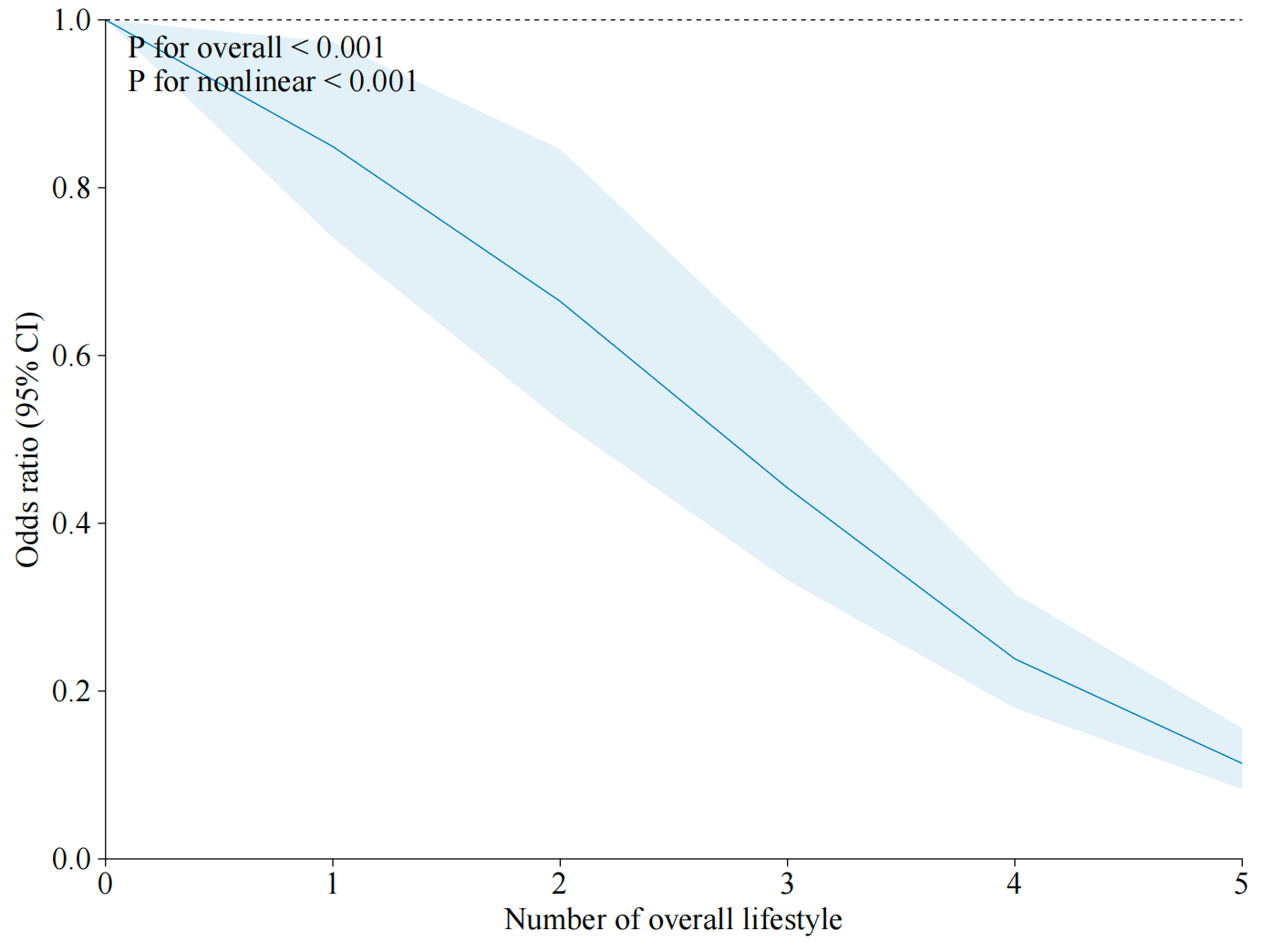


# Figure S2 Dose-relationship of the number of healthy lifestyle and odds ratio of depressive symptoms.

Graphs show ORs for depressive symptoms according to overall lifestyle adjusted for age, gender, region, household income, only child, educational level, marital status, and recent negative life events. Data were fitted by a logistic regression model, and the model was conducted with 3 knots at the 5th, 50th, 95th percentiles of lifestyle. Solid lines indicate ORs, and shadow shape indicate 95% CIs.

OR: odds ratio; CI: confidence interval.

#
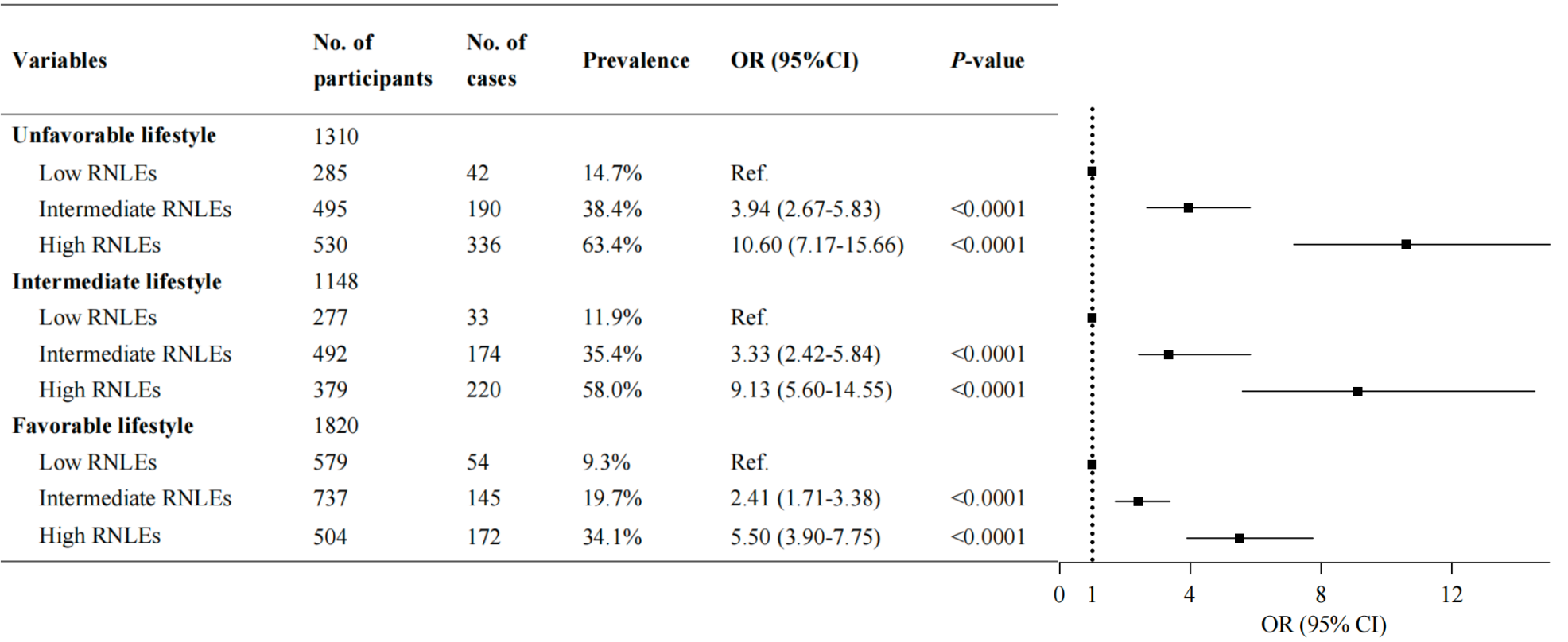


# Figure S3 Association between recent negative life events (RNLEs) and odds ratio of depressive symptoms within each overall lifestyle category.

Note: Participants with Low RNLEs were considered as the reference group within each overall lifestyle category. Points represent the odds ratios, and error bars show the 95 % confidence intervals. The model was adjusted for age, gender, region, only child, education level, household income, and marital status.


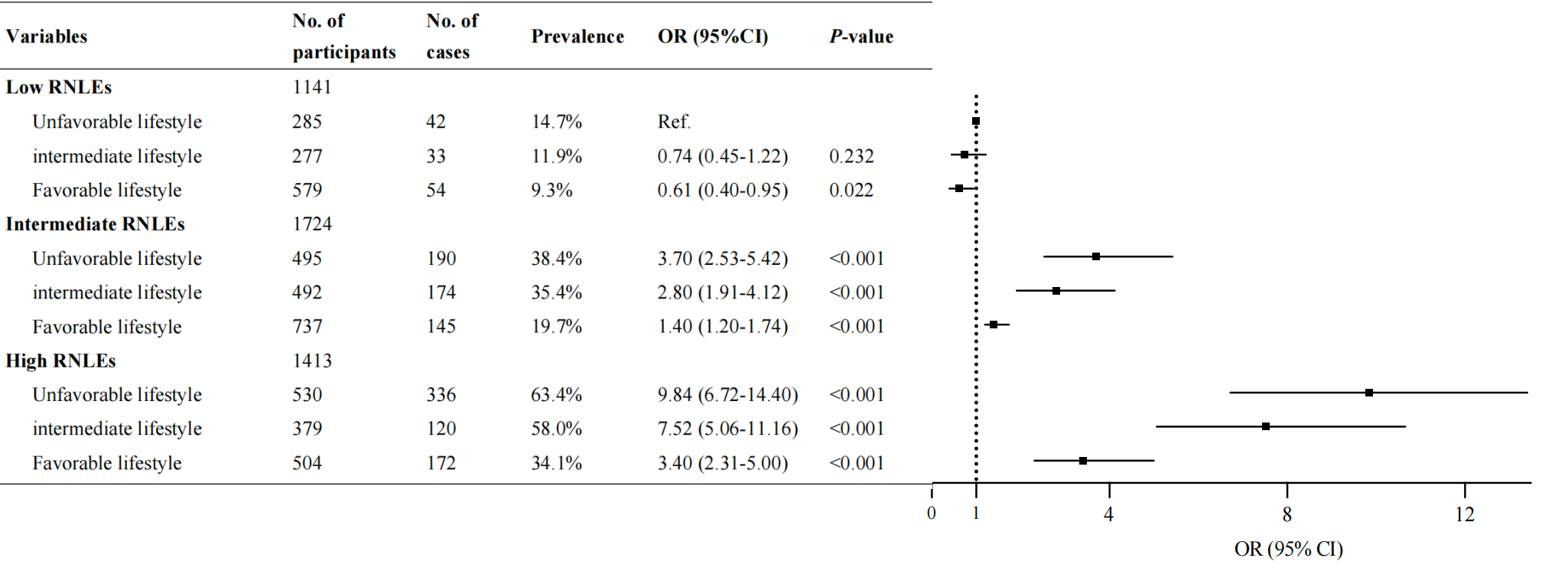


# Figure S4 Joint association of recent negative life events (RNLEs) and overall lifestyle in adulthood with odds ratio of depressive symptoms.

There was a significant interaction effect between RNLEs and overall lifestyle on depressive symptoms (P value <0.0001)
